# Age, Sex and Overall Health, Measured As Frailty, Modify Myofilament Proteins in Hearts From Naturally Aging Mice

**DOI:** 10.1038/s41598-020-66903-z

**Published:** 2020-06-22

**Authors:** Alice E. Kane, Elise S. Bisset, Kaitlyn M. Keller, Anjali Ghimire, W. Glen Pyle, Susan E. Howlett

**Affiliations:** 1 Blavatnik Institute, Department of Genetics, Paul F. Glenn Center for Biology of Aging Research at Harvard Medical School, Boston, MA US; 2 Department of Pharmacology, Dalhousie University, Halifax, NS Canada; 3 Department of Biomedical Sciences, University of Guelph, Guelph, Ontario, Canada; 4 IMPART team Canada Investigator Network, Saint John, New Brunswick, Canada; 5 Department of Medicine (Geriatric Medicine), Dalhousie University, Halifax, NS Canada

**Keywords:** Cardiovascular biology, Comorbidities

## Abstract

We investigated effects of age, sex and frailty on contractions, calcium transients and myofilament proteins to determine if maladaptive changes associated with aging were sex-specific and modified by frailty. Ventricular myocytes and myofilaments were isolated from middle-aged (~12 mos) and older (~24 mos) mice. Frailty was assessed with a non-invasive frailty index. Calcium transients declined and slowed with age in both sexes, but contractions were largely unaffected. Actomyosin Mg-ATPase activity increased with age in females but not males; this could maintain contractions with smaller calcium transients in females. Phosphorylation of myosin-binding protein C (MyBP-C), desmin, tropomyosin and myosin light chain-1 (MLC-1) increased with age in males, but only MyBP-C and troponin-T increased in females. Enhanced phosphorylation of MyBP-C and MLC-1 could preserve contractions in aging. Interestingly, the age-related decline in Hill coefficients (r = −0.816; p = 0.002) and increase in phosphorylation of desmin (r = 0.735; p = 0.010), tropomyosin (r = 0.779; p = 0.005) and MLC-1 (r = 0.817; p = 0.022) were graded by the level of frailty in males but not females. In these ways, cardiac remodeling at cellular and subcellular levels is graded by overall health in aging males. Such changes may contribute to heart diseases in frail older males, whereas females may be resistant to these effects of frailty.

## Introduction

Diseases of impaired myocardial contractile function, including heart failure, increase with age in both men and women^1^. This may not be surprising. Studies suggest that the heart undergoes maladaptive changes during normal aging that may set the stage for the development of heart failure^2^. However, a key challenge to understanding the milieu in which such diseases develop is that, while older men and women are most likely to develop heart diseases as they age^1^, current preclinical research studies typically use young, mostly male animals^3–5^. Although few experimental studies have investigated the influence of age on cardiac contractile function, emerging evidence suggests this may differ between the sexes both in humans and in animal models^6–9^. To understand the underlying reasons, it is important to identify cellular and subcellular mechanisms that are involved in cardiac aging in both sexes.

Although age modifies the heart, such changes are average responses that may not be present, or present to the same extent, in all individuals of the same age^10^. For example, on average ventricular contractility declines with age, even though some older men and women perform at the same or even at higher levels when compared to younger adults^11^. This suggests that aging is heterogenous. The concept of “frailty”, used by demographers in 1979 to describe unmeasured heterogeneity in mortality risk in people of the same age^12^, is now used to describe unmeasured heterogeneity in the risk of many age-related adverse outcomes in both humans and animals^13^. While there is no consensus definition of frailty^14^, it is clinically important as frail individuals are more likely to develop diseases, including heart diseases, than their non-frail counterparts^15^. It is possible that changes associated with cardiac aging are modified by frailty, but little is known about the impact of age and frailty on the heart, especially in females.

Frailty has been quantified clinically with many different instruments^16,17^. One common technique is to create a “frailty index”, by dividing the number of health deficits in an individual by the total number of deficits considered to produce a score between 0 and 1, where higher scores denote greater frailty^18^. We have adapted this approach to quantify the degree of frailty in aging rodents^13,19–21^. This provides a powerful new tool that can be used to explore the relationship between cardiac aging and overall health (frailty), in mice of both sexes. The goal of this study is to investigate the impact of age, sex and frailty on cardiac contractile function and explore underlying mechanisms that regulate contraction in a mouse model. Studies used isolated ventricular myocytes, Langendorff-perfused hearts and ventricular homogenates from middle-aged (~12 months) and older (~24 months) male and female C57Bl/6 mice. Frailty was evaluated in each animal with a frailty index tool that measures frailty as the accumulation of health deficits across many diverse systems, but not the cardiovascular system *per se*^22^.

## Results

### Calcium transients declined with age, but contractions were largely unaffected in both field-stimulated ventricular myocytes and intact hearts from male and female mice

**Figure 1:**
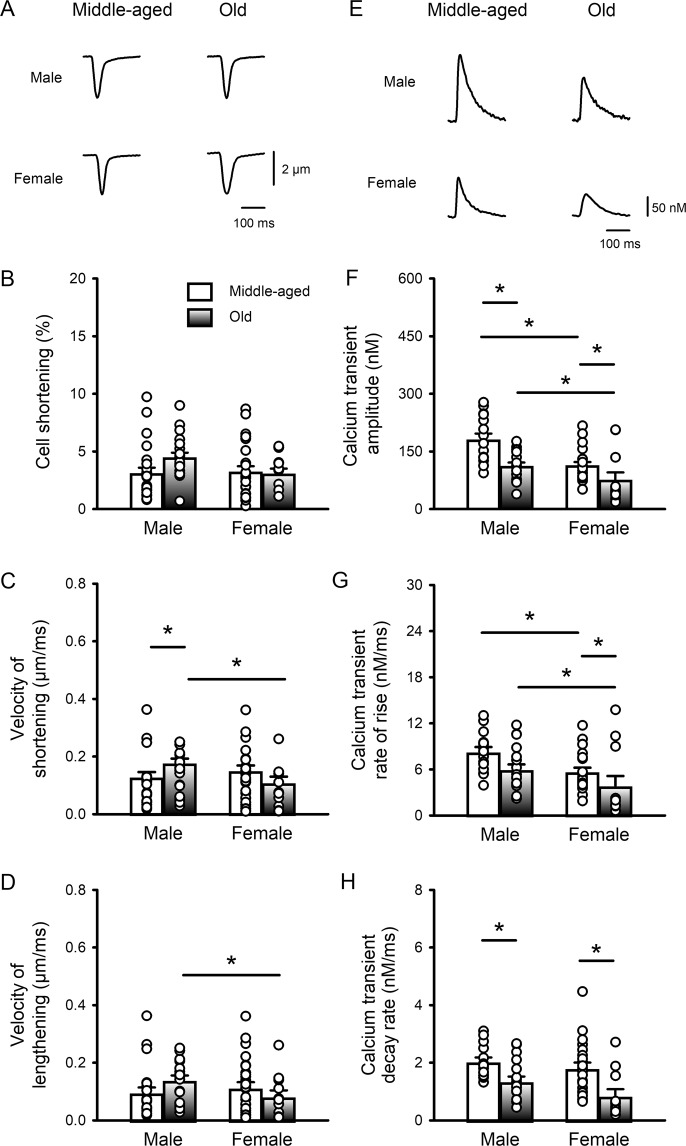
Peak calcium transients declined and slowed with age in C57BL/6 mice of both sexes, but contractions were largely unaffected. (**A**) Representative examples of contractions (cell shortening) recorded from field-stimulated (4 Hz) ventricular myocytes isolated from middle-aged (~12 mos) and older (~24 mos) male and female mice. (**B**) Mean data show that peak contractions were similar in all four groups. (**C**) The velocity of shortening increased slightly with age in males and was faster in older male cells compared to female cells. (**D**) The velocity of lengthening was unaffected by age but was lower in older females than in older males. (**E**) Representative examples of calcium transients recorded from myocytes from middle-aged and older mice of both sexes. (**F**) Mean data show that peak calcium transients declined with age in both sexes and were smaller in cells from females than males at both ages. (**G**) The rates of rise of the calcium transient declined with age and this was significant in females. The rates of rise were slower in females than males at all ages. **(H)** The rates of decay of the calcium transients declined markedly with age in both sexes. Values represent the mean ± SEM values in each case. Data were analyzed by two-way ANOVA with age and sex as main factors (post-hoc test was Holm-Sidak). The * denotes p < 0.05. For calcium transients n = 15, 18, 22 and 12 myocytes from 6 middle-aged male mice, 4 older males, 9 middle-aged females and 3 older females, respectively. For contractions n = 24, 20, 27 and 12 myocytes from 6 middle-aged male mice, 4 older males, 8 middle-aged females and 3 older females, respectively.

Initial experiments determined whether ventricular myocyte contractions and the underlying calcium transients were affected by age and whether this differed between the sexes. Figure 1A shows representative contractions recorded from ventricular myocytes (paced at 4 Hz) isolated from the hearts of middle-aged (~12 mos) and older (~24 mos) male and female mice. The mean (± SEM) data show that contraction amplitudes were similar regardless of age or sex (Fig. 1B). The speed of shortening showed a modest increase with age in males, which was significantly different from older females (Fig. 1C). The velocity of lengthening was not affected by age but was slower in older females compared to older males (Fig. 1D). These results indicate that there are few age- or sex-dependent changes in contractions in field-stimulated ventricular myocytes between middle-age and later life.

Figure 1E shows examples of calcium transients recorded from ventricular myocytes from the hearts of middle-aged and older mice. Mean data show that calcium transient amplitudes declined with age in both sexes (Fig. 1F). There also was a sex difference where, regardless of age, calcium transients were smaller in cells from females than males. We evaluated the impact of age and sex on calcium transient rates of rise and decay (Fig. 1G,H). The calcium transient rates of rise declined with age and this was significant for females, where rates of rise were slower than for males at both ages (Fig. 1G). Age was also associated with a dramatic slowing of calcium transient decay rate in both sexes (Fig. 1H). These observations show that aging was associated with an overall decrease in the magnitude and speed of calcium transients in both sexes, with few parallel changes in contractions.

**Figure 2:**
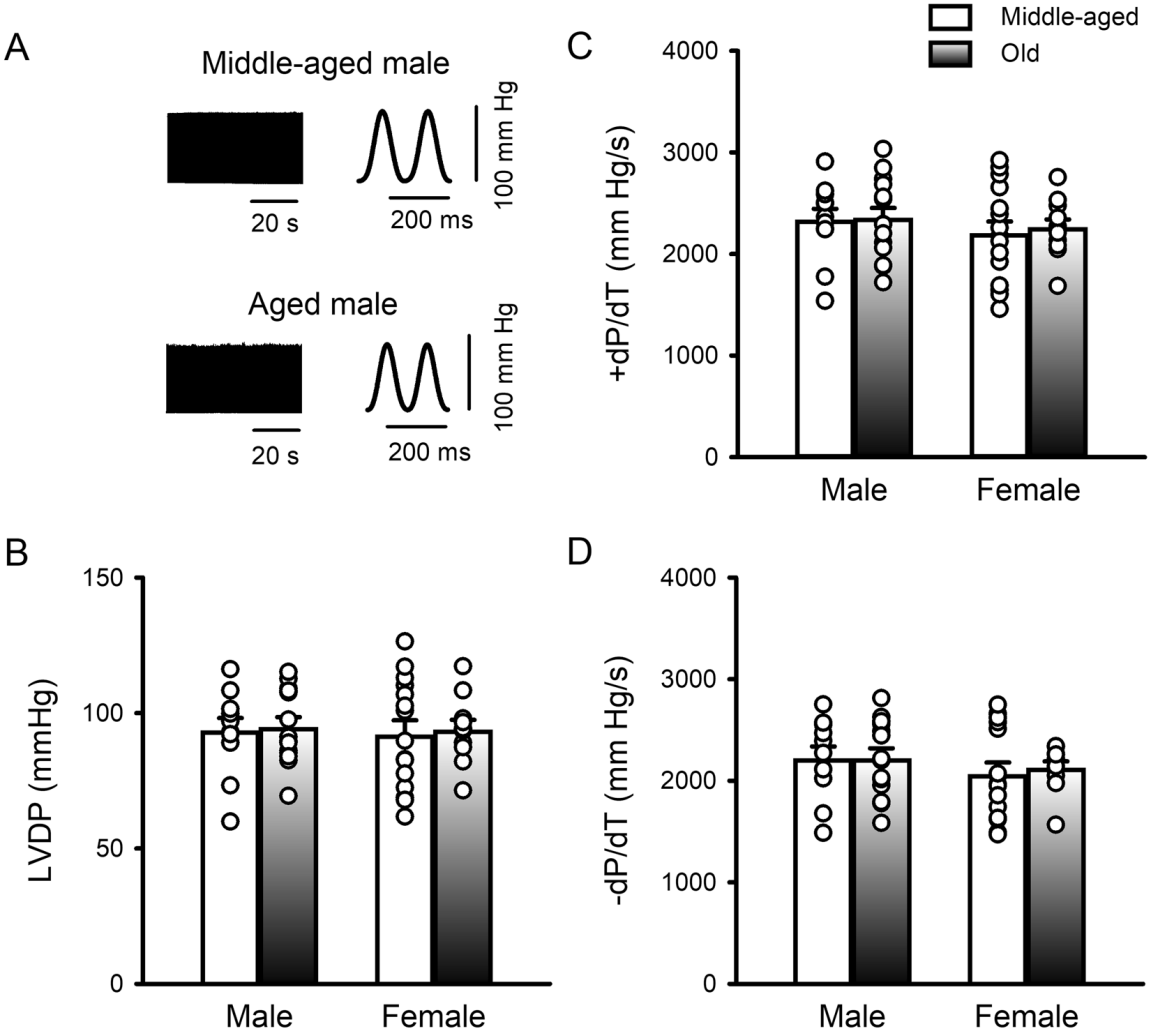
Left ventricular contractile function was similar in hearts from middle-aged and older mice, regardless of sex. (**A**) Examples of pressure recordings from Langendorff-perfused hearts isolated from middle aged and older male mice. Recordings are shown at condensed (left) and expanded (right) time scales. (**B–D**) Scatterplots demonstrate that LVDP, + dP/dt and −dP/dt were similar in hearts from middle-aged and older mice of both sexes. Data were analyzed by two-way ANOVA with age and sex as main factors (post-hoc test was Holm-Sidak). Samples were hearts from 11 middle-aged male mice, 14 older males, 15 middle-aged females and 11 older females, respectively.

To investigate whether cardiac contractile function changed between 12 and 24 months of age, we also compared left ventricular developed pressure (LVDP) in Langendorff-perfused hearts from both sexes. Figure 2A shows representative examples of LVDP recorded from isolated perfused hearts from middle-aged and older male mice. Figure 2B illustrates mean data that show that peak LVDP was similar, regardless of age or sex. Likewise, the rates of pressure development (Fig. 2C) and decay (Fig. 2D) were similar in hearts from middle-aged and older mice of both sexes. Thus, even though aging was associated with smaller, slower calcium transients, contractile function was unaffected in intact hearts and isolated myocytes. To explore potential underlying mechanisms, we next investigated whether changes in the myofilaments occurred during the aging process.

### Actomyosin Mg-ATPase activity markedly increased with age in myofilaments from female but not male hearts

**Figure 3:**
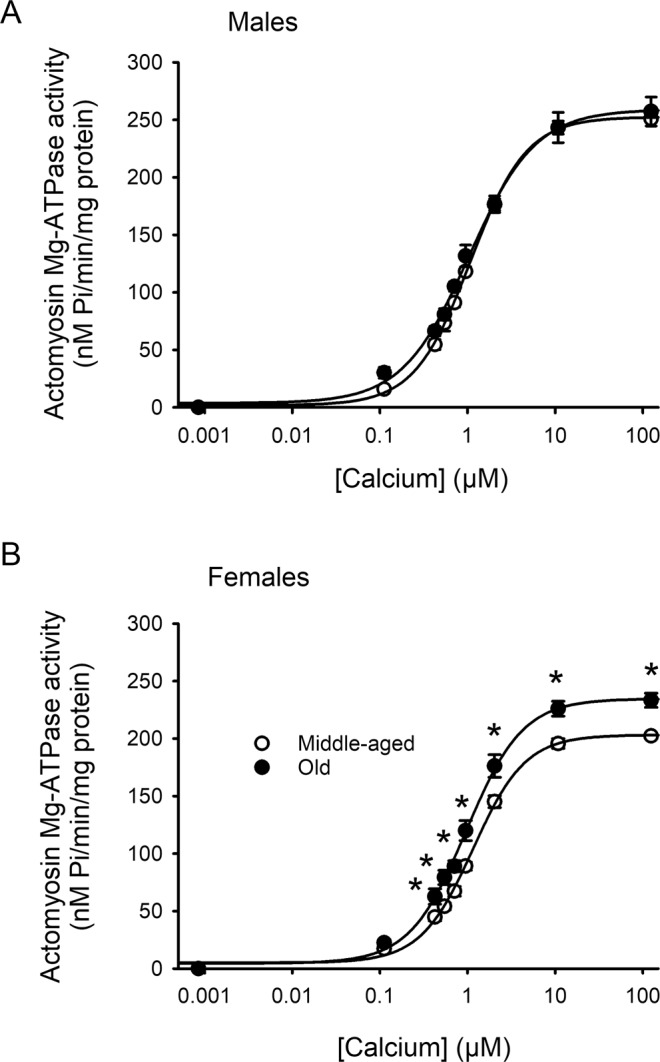
Maximal actomyosin Mg-ATPase activity increased with age in hearts from females but not males. (**A**) Actomyosin Mg-ATPase activity increased as calcium concentrations increased to the same extent in myofilaments from middle-aged and older male hearts. (**B**) In females, actomyosin Mg-ATPase activity increased with age at almost all calcium concentrations tested. Values represent the mean ± SEM values. Data were analyzed with a two-way repeated measures ANOVA, with age as the main factor, calcium concentration as the repeated measure and pairwise multiple post-hoc comparisons with a Tukey test. The * symbol indicates a significant effect of age. Values were significant for p < 0.05. Samples were hearts from 5 middle-aged male mice, 6 older males, 5 middle-aged females and 5 older females, respectively.

**Figure 4:**
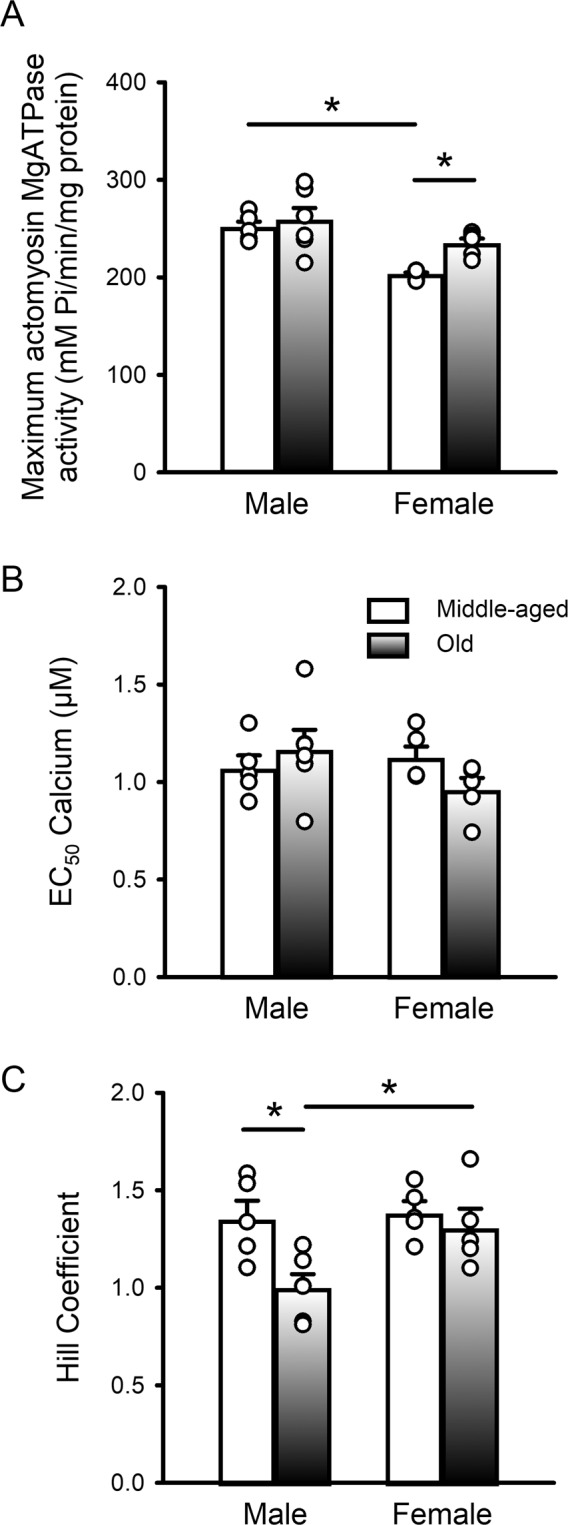
Maximal actomyosin Mg-ATPase activity and Hill coefficients varied with age in a sex-specific fashion, but EC_50_ values were unaffected. (**A**) Mean data show that maximal actomyosin ATPase activity increased with age in females but not males. There was also a sex difference at younger ages where activity was higher in middle-aged males than in middle-aged females. (**B**) EC_50_ values were similar in all four groups. (**C**) Average values for the Hill coefficients declined markedly with age in males and were significantly lower in older males compared to older females. Values denote the mean ± SEM in each case. Data were analyzed with a two-way ANOVA with age and sex as main factors (post-hoc test was Holm-Sidak). The * denotes p < 0.05. Samples were hearts from 5 middle-aged male mice, 6 older males, 5 middle-aged females and 5 older females, respectively.

We investigated the relationship between activating calcium concentrations and actomyosin Mg-ATPase activity in myofilaments isolated from male and female ventricles at both ages. Results are shown in Fig. 3. When absolute actomyosin Mg-ATPase activity was plotted as a function of calcium in males, there was no difference between middle-aged and older hearts (Fig. 3A). By contrast, actomyosin Mg-ATPase activity increased with age in females, and this increase was statistically significant at physiologically relevant calcium levels above ~500 nM free calcium (Fig. 3B). We also compared maximal actomyosin Mg-ATPase activity between all four groups, as shown in Fig. 4A. The average maximal actomyosin Mg-ATPase activity increased with age in females but not males. Interestingly, activity was lowest in middle-aged females and was significantly lower than age-matched males (Fig. 4A). These data demonstrate that there was an increase in actomyosin Mg-ATPase activity with age across a wide range of activating calcium concentrations, although this effect was sex-specific and occurred only in hearts from females.

When the actomyosin Mg-ATPase activity data were normalized to the maximum for each group, there were no significant age or sex effects (Supplementary Figure 1A,B). Consistent with this finding, the average EC_50_ values (concentration of calcium required to produce 50% activation), did not differ between groups (Fig. 4B). The steepness of the actomyosin Mg-ATPase-calcium relationship is indicated by the Hill coefficient such that larger values denote increased cooperativity of calcium activation. To determine if there were age- or sex-related changes in the steepness of the actomyosin Mg-ATPase versus calcium curves, we compared mean Hill coefficients between groups. On average, Hill coefficients declined markedly with age in males, although there was heterogeneity and individual data points for the two age groups overlapped (Fig. 4C). By contrast, there was no age-dependent change in females, but Hill coefficients were significantly higher in older females when compared to older males (Fig. 4C). These findings indicate that cooperativity of the actomyosin Mg-ATPase-calcium relationship declined with age in male hearts.

### Overall health, measured with a frailty index, graded age-related changes in myofilament function in males but not females

**Figure 5:**
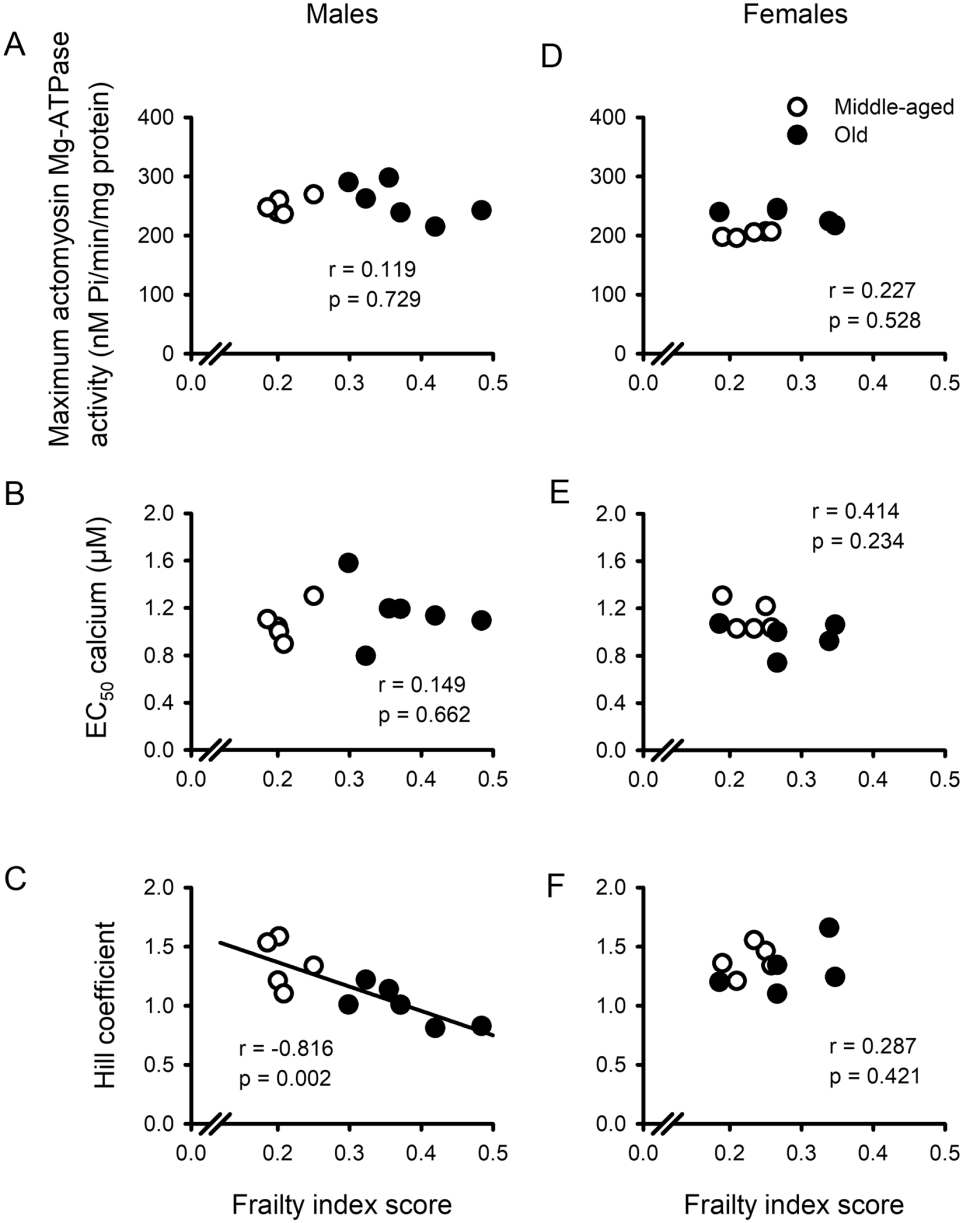
The Hill coefficients for the actomyosin Mg ATPase activity versus calcium curves were graded by frailty in males only. (**A,D**) Maximal actomyosin Mg-ATPase activity was not related to frailty scores in either males or females. (**B,E**) EC_50_ values also were not correlated with frailty index scores in either sex. (**C,F**) In contrast, Hill coefficients were graded by the level of frailty, but only in males. Data were fitted with a regression line. Correlation coefficients and p values are indicated on each panel and a line was drawn for statistically significant correlations (p < 0.05). Samples were hearts from 5 middle-aged male mice, 6 older males, 5 middle-aged females and 5 older females, respectively.

On average, Hill coefficients declined with age in males (Fig. 4C) whereas maximal actomyosin Mg-ATPase activity increased with age in females (Fig. 4A). However, there was considerable heterogeneity, especially for the Hill coefficients, such that individual values from the two age groups overlapped in many cases. To determine the relationship between parameters derived from the actomyosin Mg-ATPase activity curves and frailty, we plotted maximal actomyosin Mg-ATPase activity, EC_50_ values and Hill coefficients as a function of frailty index score (Fig. 5). We fitted each curve by linear regression. We found that there was no correlation between maximal actomyosin Mg-ATPase activity and frailty index scores in either males (Fig. 5A) or females (Fig. 5D). Similarly, EC_50_ values were not correlated with the level of frailty in either sex (Fig. 5B,E). By contrast, Hill coefficients, which declined with age in males only, exhibited a strong negative association with frailty in males (Fig. 5C) but showed no relationship with frailty in females (Fig. 5F). As both age and frailty were related to the decline in Hill coefficients in male hearts, we used multivariable regression to calculate the semi-partial correlations to assess the separate contributions of age and frailty to this relationship. With respect to the semi-partial correlations, we found that both frailty (r = −0.816) and age (r = −0.726) contributed significantly to the decline in Hill coefficients. This indicates that age-dependent changes in actomyosin Mg-ATPase activity in male hearts were graded by the overall health of the animal, as quantified with a frailty index score.

### Male and female hearts exhibited distinct age-associated changes in myofilament protein phosphorylation

**Figure 6:**
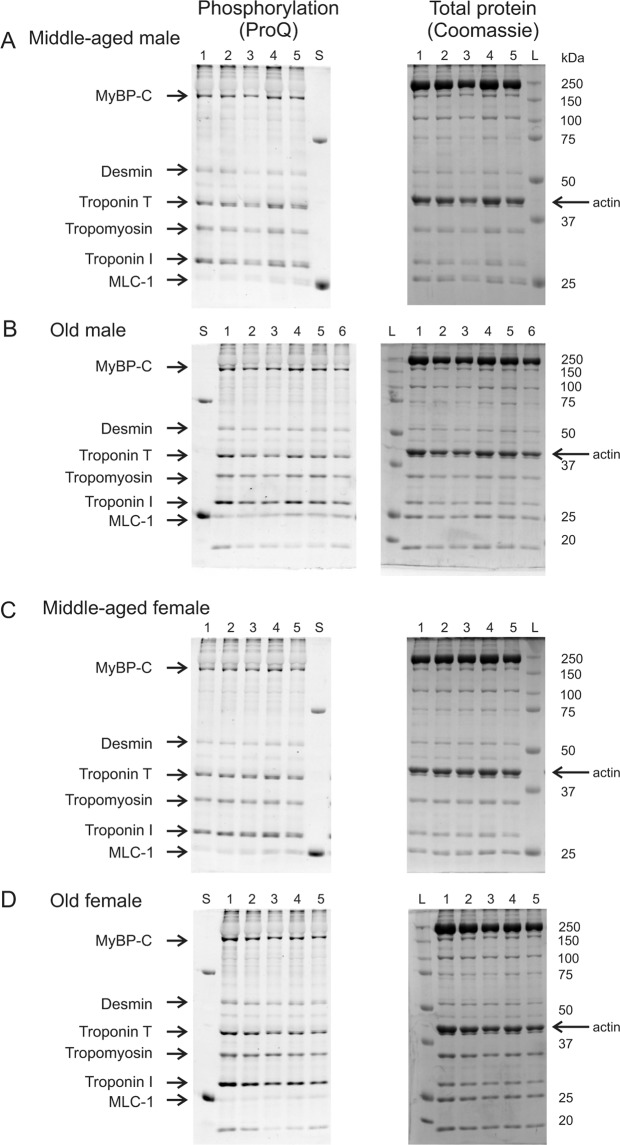
Data illustrating the protein phosphorylation of major myofilament proteins in hearts from middle-aged and older males and females of both sexes. Gels illustrate phosphorylation staining (ProQ Diamond) and total protein staining (Coomassie blue) for all samples quantified in this study. Individual samples in each group are indicated numerically at the top of each lane. Protein standards at 25 and 75 kDa are visible in the ProQ images (lane labelled “S”) and the molecular weight ladder is visible in the Coomassie images (lane labelled “L”). Actin (42 kDa) was used as a loading control and is shown with an arrow on each Coomassie image. (**A,B**) Myofilament proteins from middle-aged and older male ventricles were separated on SDS-PAGE and stained with Pro-Q (left) to compare phosphorylation. They were also stained with Coomassie blue (right) to illustrate protein load. (**C,D**) Myofilament proteins from middle-aged and older females separated by Pro-Q (left) and stained with Coomassie (right). Major myofilament proteins are indicated on the left side of each panel and molecular weight markers (kDa) are shown on the right. Myosin-binding protein C (MyBP-C), myosin light chain-1 (MLC-1). The gels have been cropped to the edges; the uncropped gels are included as Supplementary Information Files 1–3. Samples were hearts from 5 middle-aged male mice, 6 older males, 5 middle-aged females and 5 older females, respectively.

**Figure 7:**
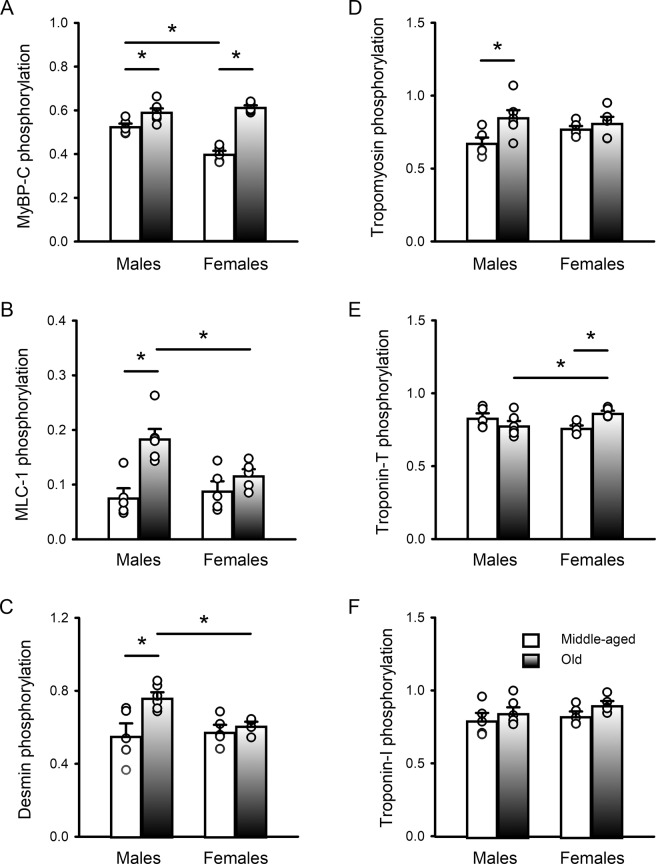
Total phosphorylation levels for MyBP-C, MLC-1, desmin & tropomyosin increased with age in males, while only MyBP-C and troponin-T increased with age in females. (**A**) MyBP-C phosphorylation increased moderately with age in males and dramatically with age in females; MyBP-C phosphorylation also was greater in middle-aged males compared to females. (**B**) MLC-1 phosphorylation increased markedly with age in males but not females; values were higher in older males than age-matched females. (**C**) Desmin phosphorylation increased with age in males only; values were significantly higher in older males compared to older females. (**D**) Tropomyosin phosphorylation increased with age in males only. (**E**) Phosphorylation of troponin-T increased with age in females only and was significantly higher in older females compared to older males. (**F**) Phosphorylation of troponin-I was not affected by either age or sex. Values denote the mean ± SEM in each case. Data were analyzed with two-way ANOVA (age and sex were main factors; the post-hoc test was Holm-Sidak). The * denotes p < 0.05. Samples were hearts from 5 middle-aged male mice, 6 older males, 5 middle-aged females and 5 older females, respectively. Actin was used as a loading control; it was not affected by either age or sex (not shown). In all cases data were normalized to actin. Myosin-binding protein C (MyBP-C), myosin light chain-1 (MLC-1).

As changes in the phosphorylation of key myofilament proteins could modify contractile function, we compared myofilament phosphorylation patterns in hearts from middle-aged and older mice of both sexes. Figure 6A–D shows gels of myofilament proteins for all samples evaluated in this study. The proteins were stained with Pro-Q (left) to visualize total phosphorylation levels and stained with Coomassie blue (right) to indicate total protein load. Actin was used as a loading control and the uncropped gels are shown in Supplementary Information Files 1–3. The mean data as well as scatterplots of the individual data points are shown in Fig. 7. Results show that, on average, myosin binding protein-C (MyBP-C) phosphorylation increased with age in both sexes (Fig. 7A). There was also a sex difference where MyBP-C phosphorylation was greater in middle-aged males than age-matched females. Mean phosphorylation levels for myosin light chain-1 (MLC-1), desmin and tropomyosin all increased with age in males but there were no age-associated changes in females (Fig. 7B–D). In all cases, there was heterogeneity in data, especially for the older males, such that values from the two different age groups showed considerable overlap. There were also sex differences in the older group, where MLC-1 and desmin phosphorylation were higher in older males than older females (Fig. 7B,C). In contrast, troponin-T phosphorylation increased with age in females only and was higher in older females than in older males (Fig. 7E). Phosphorylation of troponin-I was not affected by age or sex (Fig. 7F). These experiments demonstrate that, on average, phosphorylation of MyBP-C, MLC-1, desmin and tropomyosin increased with age in males, but only MyBP-C and troponin-T phosphorylation increased with age in females.

### Age-related changes in myofilament phosphorylation were graded by frailty index scores in males but not females

**Figure 8:**
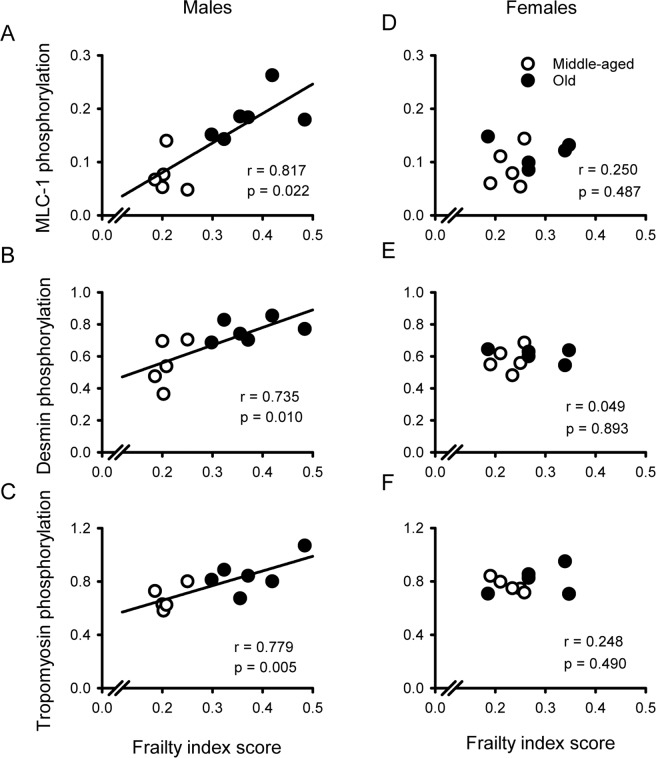
Phosphorylation of the key myofilament proteins MLC-1, desmin and tropomyosin were highly correlated with overall health, as indicated by frailty index scores, in hearts from males only. (**A–C**) The phosphorylation of desmin, MLC-1 and tropomyosin exhibited strong positive correlations with frailty in hearts from males. (**D–F**) Although phosphorylation levels for many myofilament proteins increased as frailty scores increased in males, there were no correlations between myofilament protein phosphorylation and frailty in hearts from females. The data were fit with a regression line. Correlation coefficients plus p values are indicated on each panel; lines were drawn for all statistically significant correlations (p < 0.05). Samples were hearts from 5 middle-aged male mice, 6 older males, 5 middle-aged females and 5 older females, respectively. Myosin light chain-1 (MLC-1).

We found that phosphorylation of several key myofilament proteins increased with age, especially in hearts from males (Fig. 7). Still, there was considerable heterogeneity, especially in data from the older males where values from the two age groups exhibited substantial overlap. To determine the relationship between myofilament protein phosphorylation and frailty, we plotted phosphorylation levels as a function of frailty and fitted the curves by linear regression as shown in Fig. 8. Results showed that phosphorylation levels for MLC-1, desmin and tropomyosin exhibited strong positive correlations with frailty in hearts from males (Fig. 8A–C). When we examined the semi-partial correlations, we found that both frailty (r = 0.817) and age (r = 0.828) contributed significantly to the higher phosphorylation levels for MLC-1 in males (Fig. 8A). Frailty and age also contributed significantly to higher phosphorylation levels for desmin (r = 0.735 for frailty and r = 0.724 for age) and tropomyosin (r = 0.779 for frailty and r = 0.645 for age; Fig. 8B,C). Unlike males, there were no correlations between phosphorylation of MLC-1, desmin or tropomyosin and frailty index scores in hearts from females (Fig. 8D–F). Phosphorylation levels for MyBP-C, troponin-T and troponin-I were not related to frailty scores in either sex (Supplementary Figure 2A–F). Together these results show that phosphorylation of key myofilament proteins increased as frailty increased in hearts from males but not females. This indicates that age-dependent increases in the phosphorylation of key myofilament proteins were graded by overall health, as quantified in a frailty index, but only in hearts from male animals.

## Discussion

This study evaluated the impact of age, sex and frailty on cardiac contractile function and explored underlying mechanisms that regulate contraction in the murine heart. Studies in isolated ventricular myocytes showed that calcium transients declined and slowed between middle age and later life in both sexes. By contrast, contractions in myocytes and in intact hearts were relatively unaffected by age. Myofilament analysis showed that actomyosin Mg-ATPase activity increased with age at physiological calcium concentrations in hearts from females, whereas myofilament cooperativity as represented by Hill coefficients declined in males. Age was accompanied by changes in the phosphorylation levels of several major myofilament proteins. However, the patterns of change differed between the sexes, and age effects were much more prominent in males. These age-associated changes in cooperativity and myofilament phosphorylation were correlated with and graded by the level of frailty in males. By contrast, no relationship between frailty and the myofilament parameters measured in this study was seen in females. Our work highlights the substantial heterogeneity in the impact of age on myofilament proteins, especially in male hearts, and show that both frailty and chronological age contribute significantly to this variance. These observations suggest that differences in overall health status contribute importantly to the impact of age on the heart. Even so, these modifications are sex-specific and are most apparent at high levels of frailty in males only.

The results of the present study showed that age had no effect on peak contractions recorded from field-simulated ventricular myocytes from mice of both sexes. This agrees with results of previous studies in field-stimulated ventricular myocytes from aged rodents when compared to young adult males^23–25^ and females^26,27^. Here, we have extended these observations to show that contractions did not change between middle age and later life, even though calcium transients declined and slowed in field-stimulated cells from mice of both sexes. We also confirmed that baseline contractile function in isolated intact hearts did not change between middle-age and later life regardless of sex^28^. Given that cardiac contraction is proportional to the magnitude of intracellular calcium release^29^, our finding that calcium transients decline with age and contractions do not is unexpected.

To explore mechanisms that might maintain contractile performance in aging in the face of reduced calcium availability, we examined myofilament calcium sensitivity. Previous work showed that myofilament calcium sensitivity declined when 2–4-month-old mice were compared to 2-year-old animals, although this study used only males^25^. Prior studies of sex differences in myofilament calcium sensitivity used young animals only and found either no sex difference^30^ or lower calcium sensitivity in hearts from males when compared to females^31^. To our knowledge, the present study is the first to investigate sex-dependent changes in myofilament calcium sensitivity in the setting of aging. We found that submaximal actomyosin Mg-ATPase activity increased markedly with age, but this was seen in female hearts only. Critically, higher actomyosin Mg-ATPase activity occurred in females at calcium concentrations within the normal physiological range^32^. Our observation that the influence of age on myofilaments is sex-specific is a key finding. The increase in myofilament calcium sensitivity in the aging female heart is likely to be compensatory and may help preserve contractile function in the face of lower intracellular calcium availability in aging.

How enhanced myofilament calcium sensitivity arises in older females is not yet clear. One possibility is that the low circulating estradiol levels seen in older female mice and rats^33^ could be involved. In support of this, studies in young, ovariectomized females show that short-term exposure to low circulating estradiol increases myofilament calcium sensitivity in the heart^34,35^. In addition, higher actomyosin Mg-ATPase activity occurs early in a murine model of menopause in which ovarian function is gradually reduced by 4‐vinylcyclohexene diepoxide injections^36^. On the other hand, both long-term ovariectomy and longer-term exposure to menopause appear to reduce cardiac myofilament calcium sensitivity in the mouse model^36,37^. Thus, whether low circulating estradiol levels can explain enhanced myofilament calcium sensitivity in naturally aging animals is not clear and may be dependent on the duration of the estradiol deficiency; additional experiments that explore this question would be of interest.

We also found that age had very little effect on actomyosin Mg-ATPase activity in males, so other regulatory mechanisms that could maintain contraction in the face of lower calcium availability were explored. As age reduces circulating testosterone levels in older mice^33^, the present results suggest that low testosterone may have little effect on myofilament calcium sensitivity. This agrees with our earlier work in male mice where we showed that gonadectomy did not influence myofilament calcium sensitivity at physiological calcium levels^38^. However, the present study did show that Hill coefficients declined with age in males. The Hill coefficient represents the steepness of the actomyosin Mg-ATPase-calcium relationship, where a larger value indicates positive cooperativity of calcium activation^39^. Cooperativity has been attributed to a variety of mechanisms, all of which involve tropomyosin either directly or indirectly^39^. Interestingly, we found that phosphorylation of tropomyosin increased with age in males but not females. This may influence the degree of cooperativity observed in aging male hearts. In support of this mechanism, there is evidence that cooperativity declines when tropomyosin is phosphorylated in reconstituted cardiac muscle fibres^40^. Thus, it is possible that enhanced phosphorylation of tropomyosin reduces the cooperativity of calcium activation. However, this change is likely to disrupt rather than preserve contractile function in the aging male heart.

We observed sex-specific changes in phosphorylation of myofilament proteins with age. For example, hearts from older males exhibited an increase in the phosphorylation of both desmin and MLC-1. Desmin is a myofilament protein that provides a scaffold to preserve cardiac myocyte structure and protect the heart from stressors such as mechanical stress^41^. Phosphorylation of desmin is thought to facilitate desmin misfolding, which may generate toxic protein aggregates that have been implicated in the pathogenesis of diseases such as heart failure^42^. The dysfunctional role of desmin phosphorylation may be mediated through the accumulation of these amyloid-like oligomers, which increase cytoskeletal cell stiffness and decrease cytoskeletal viscosity thereby presenting a physical impediment to contractility^42^. Thus, elevated desmin phosphorylation may be maladaptive in the aging male heart. We also observed increased MLC-1 phosphorylation in aging male hearts. Although relatively little is known about its role in cardiac contraction, hypophosphorylation of MLC-1 in a zebra fish model disrupts myocardial force generation and increases the heart’s susceptibility to stress^43^. Thus, this increase in MLC-1 phosphorylation may represent a beneficial effect to compensate for deleterious effects of desmin hyperphosphorylation in aging male hearts. We also found a significant increase in troponin-T phosphorylation in aging females but the significance of this is unclear. Although troponin-T can be phosphorylated at several different sites, this appears to have little impact on myofilament function^44,45^.

Our work showed that MyBP-C phosphorylation increased with age in both sexes. MyBP-C regulates cardiac contractile function and is controlled by phosphorylation through multiple signaling pathways, although its contributions to heart function are not fully understood^46^. Interestingly, dephosphorylation of cardiac MyBP-C is a common finding in diseases of contractile dysfunction, such as heart failure, in both humans and animal models^47,48^. In addition, transgenic mice with non-phosphorylatable MyBP-C exhibit impaired systolic and diastolic function^49^. Together these findings indicate that chronic dephosphorylation of MyBP-C disrupts myocardial contractile function. As phosphorylation of cardiac MyBP-C induces a conformational change in myosin that promotes cross-bridge formation^50^, an increase in MyBP-C phosphorylation may be a compensatory mechanism that helps preserve cardiac contractile function in aging. While MyBP-C phosphorylation increased with age and maybe be a compensatory mechanism in both sexes, opposing phosphorylation changes that impair myofilament function (*e.g*. desmin phosphorylation) in males may off-set beneficial alterations in the contractile apparatus.

Frailty is a key determinant of overall health status and mortality in mice of similar chronological ages, as it is in humans^13^. We have previously shown that age-associated adverse remodeling in the atria and ventricles is graded by the level of frailty in male mice^10,51,52^. This suggests that cardiac aging and overall health are closely linked, at least in males. A novel and important finding in the present study is our observation that, while most age-associated changes seen in male hearts were graded by frailty, none of the age-dependent changes in females exhibited any clear relationship with frailty. As identified by Maric-Bilkan *et al*.^53^, it is critically important to conduct basic research in cardiovascular biology in animals of both sexes to address the knowledge gap in how sex can affect research outcomes. Our results highlight the importance of using both male and female animals in such studies, as very different conclusions would have been reached had only one sex been used here. Taken together, our data suggest that poor overall health, quantified in a frailty index, predicts adverse changes and post-translational modifications in myofilaments in the aging male heart but not in the aging female heart. Some of these changes, such as the increased phosphorylation of MLC-1 at high frailty levels, may be compensatory and help preserve contractile function in hearts from frail older men. Still, enhanced phosphorylation of desmin and tropomyosin, as well as smaller Hill coefficients, would be expected to negatively affect heart function and may ultimately promote the development of diseases of impaired contractility in frail older men. Our findings also suggest that females may be more resilient than males to the effects of poor overall health on the heart.

We have demonstrated that intracellular calcium availability declined between middle age and later life in myocytes from male and females, but contractions were preserved. Contractile function was maintained in aging females by an increase in myofilament calcium sensitivity and enhanced phosphorylation of MyBP-C. By contrast, there was no compensatory increase in myofilament calcium sensitivity in males, although enhanced phosphorylation of MLC-1 and MyBP-C in males may help preserve contractile function in aging. Still, the elevated phosphorylation of tropomyosin and desmin, as well as the decrease in positive cooperativity, would be expected to ultimately impair contractile function in older males. We found that the impact of age on myofilament proteins was heterogenous in male hearts and our results demonstrate that both frailty and chronological age contribute significantly to this variance. This suggests that there is a link between cardiac aging and overall health in aging males, while older females may be resistant to the adverse effects of frailty on the heart. These findings may help explain the so-called morbidity-mortality paradox, where older women have higher levels of frailty than men at any age, but live longer^54^. Further exploration of the mechanistic basis for sex-specific changes in aging and frailty is motivating additional inquires by our group.

## Methods

### Animals

C57BL/6 mice of both sexes were purchased from Charles River (St. Constant, QC, Canada) and aged in the Carlton Animal Care Facility at Dalhousie University until use. Mice were housed in groups in individually ventilated caging systems (Allentown Inc; 21 °C; 35% humidity). They were kept on a 12-hour light/dark cycle with free access to water and food (ProLab RMH 3000, Purina LabDiet, Aberfoyle, Ontario, Canada). Animal protocols were approved by the Dalhousie University Committee on Laboratory Animals and studies were performed in accordance with the guidelines of the Canadian Council on Animal Care (CCAC, Ottawa, ON: Vol 1, 2nd edition, 1993; revised March 2017).

### Mouse clinical frailty assessment

Overall health was assessed with a mouse clinical frailty index tool. This instrument is an index of 31 potential deficits in health that can accumulate with age in C57BL/6 mice^20^. This tool evaluates deficits in overall health (*e.g*. the integument, musculoskeletal system, vestibulocochlear/auditory systems, ocular/nasal systems, digestive system, urogenital system, respiratory system, signs of discomfort, body weight and body surface temperature) and none of the potential deficits is a measure of cardiovascular health *per se*. This non-invasive instrument is validated and reliable, as described previously^55,56^. Mice were assessed in a quiet room after they had acclimatized for approximately 10 minutes. They were individually evaluated for the presence of 31 potential deficits. For each item, mice without the deficit received a score of 0, those with a mild deficit received 0.5, and those with a severe deficit scored a 1. Values for each deficit were then added and divided by the total number of deficits assessed to produce a frailty index score that could theoretically be between 0 and 1.

### Field stimulation experiments

Ventricular myocytes were enzymatically dissociated with our established techniques^26^. Mice were weighed and anesthetized (200 mg/kg sodium pentobarbital IP, 100 U heparin IP). The heart was removed and perfused through the aorta at 2 mL/min (10 mins) with calcium-free buffer (mM): 105 NaCl, 5 KCl, 25 HEPES, 0.33 NaH_2_PO_4_, 1.0 MgCl_2_, 20 glucose, 3.0 Na-pyruvate, and 1.0 lactic acid (pH 7.4; 37 °C; 100% O_2_). Then the heart was perfused with this buffer plus calcium (50 μM), collagenase (8 mg/30 ml, Worthington Type I, 250 U/mg), dispase II (3.5 mg/30 ml, Roche) and trypsin (0.5 mg/30 ml, Sigma). After 8–10 minutes, the ventricles were removed, minced and stored in high potassium buffer (mM): 50 L-glutamic acid, 30 KCl, 30 KH_2_PO_4_, 20 taurine, 10 HEPES, 10 glucose, 3 MgSO_4_, and 0.5 EGTA (pH 7.4; 21 °C). Cell suspensions were filtered with a 225 μm polyethylene mesh filter (Spectra/Mesh).

Cells were loaded with calcium-sensitive dye (fura-2 AM, 2.5 μM; Invitrogen, Burlington, ON) for 20 minutes in the dark on the stage of an inverted microscope (Nikon Eclipse TE200, Nikon Canada, Mississauga, ON). Cells were superfused (3 mL/min) with buffer (mM): 145 NaCl, 10 glucose, 10 HEPES, 4 KCl, 1 CaCl_2_, and 1 MgCl_2_ (pH 7.4) at 37 °C. Cell shortening and calcium transients were simultaneously recorded by splitting the microscope light between the camera (model TM-640, Pulnix America) and photomultiplier tube (PTI, Brunswick NJ, USA) with a dichroic mirror (Chroma Tech. Corp. Rockingham, VT). Cells were viewed on a closed-circuit television monitor linked to a video edge detector (Crescent Electronics, Sandy, UT) to measure cell length (120 samples/sec). Calcium transients were measured with a DeltaRam fluorescence system and Felix software (Photon Technologies International, Birmingham, NJ). Cells were alternately excited at 340 and 380 nm and fluorescence emission at 510 nm was recorded for both wavelengths (200 samples/sec). Recordings were background corrected and emission ratios were converted to calcium concentrations with an *in vitro* calibration curve as in our earlier studies^57,58^. Cells were field-stimulated at 4 Hz with bipolar pulses delivered through platinum electrodes via a stimulus isolation unit (Model # SIU-102; Warner Instruments, Hamden, CT) controlled by pClamp 8.1 software (Molecular Devices, Sunnyvale, CA).

### Langendorff-perfused heart studies

Langendorff-perfused heart studies were conduced as we have previously described^33^. In brief, mice were weighed and anesthetized as described above. Hearts were excised, cannulated on a Langendorff apparatus (Radnoti LLC, Monrovia, Ca, USA) and perfused at constant pressure (80 ± 0.5 mmHg; 37 °C) with the following buffer (mM): 126 NaCl, 0.9 NaH_2_PO4, 4 KCl, 20 NaHCO_3_, 0.5 MgSO_4_, 5.5 glucose, and 1.8 CaCl_2_ (95% O_2_, 5% CO_2_; pH 7.4). A fluid filled balloon was inserted into the left ventricle via the left atrium and inflated to 5–10 mmHg. Pressure was recorded with a pressure transducer and PowerLab 8/35 data acquisition system (ADInstruments, Colorado Springs, CO, USA). Data were analyzed with LabChart 7 software (ADInstruments). Left ventricular pressure was measured to quantify LVDP and the maximum rates of pressure development (+dP/dt) and decay (−dP/dt). Hearts were allowed to stabilize for 20–30 minutes before recordings were made; responses over a 10-minute period were averaged.

### Myofilament studies

Myofilaments were isolated with our established techniques^59^. Briefly, mice were anesthetized with sodium pentobarbital (described above) and their hearts were removed. The ventricles were weighed, flash frozen in liquid nitrogen and stored at -80 °C. Tissue was homogenized in ice-cold buffer (mM): 60 KCl, 30 imidazole (pH 7.0), 2 MgCl_2_, 0.01 leupeptin, 0.1 PMSF, 0.2 benzamidine, and phosphatase inhibitors (P0044, Sigma-Aldrich) and centrifuged (14,000 g; 15 min; 4 °C). The pellet was re-suspended in the homogenizing buffer supplemented with 1% Triton X-100 (45 min, on ice). This solution was then centrifuged (1,100 g; 15 min; 4 °C) and the myofilament pellet was washed three times in ice-cold buffer and re-suspended in homogenizing buffer. Myofilaments were either flash frozen (for subsequent myofilament protein phosphorylation assays) or kept on ice and used immediately to assess actomyosin MgATPase activity. Myofilaments (25 µg) were incubated in ATPase buffers supplemented with increasing concentrations of free calcium (10 min; 32 °C) to quantify actomyosin MgATPase activity, as we have previously described^60^. Reactions were quenched with 10% trichloroacetic acid and then equal volumes of FeSO_4_ (0.5%) and ammonium molybdate (0.5%) in 0.5 M H_2_SO_4_ were added. The production of inorganic phosphate was measured as the absorbance at 630 nm.

Myofilament protein phosphorylation was assessed with our established techniques^59^. Briefly, myofilament proteins (10 µg) were separated with SDS-PAGE (12%) and fixed in 50% methanol-10% acetic acid (23 °C) overnight. ProQ Diamond staining was used to assess myofilament protein phosphorylation (Molecular Probes, Eugene, OR). Gels were imaged with a Bio-Rad ChemiDoc MP Imaging System (Bio-Rad Laboratories Ltd., Mississauga, ON) and they were analyzed with ImageJ (NIH, Bethesda, MD, USA). The protein load of each gel was determined by Coomassie staining, after the ProQ Diamond staining and imaging. Actin was selected to represent protein load as we have done previously^38^. To permit comparisons across gels, an equal amount of protein standard was loaded in multiple lanes of each gel. The protein standards (Bio-Rad 161-0374) at 25 and 75 kDa are visible during ProQ Diamond imaging, allowing for standardization across all gels. These standards showed equal fluorescence across all gels (<3% variation at most).

### Statistics

Data are expressed as mean ± SEM unless otherwise indicated. The effects of age and sex on each outcome were compared with a 2-way ANOVA followed by Holm-Sidak post-hoc tests. When actomyosin Mg-ATPase activity was plotted as a function of calcium concentration, male and female groups were analyzed separately with a two-way repeated measures ANOVA, with age as the main factor and calcium concentration as the repeated measure and pairwise multiple post-hoc comparisons with a Tukey test. We evaluated relationships between various parameters and frailty with linear regression analysis. When both age and frailty were significantly related to a parameter under study, we used multivariable regression and calculated semi-partial correlations to assess their separate contributions. Sigmaplot software (v15.0, Systat Software Inc.) and SPSS software (v21.0) were used for all statistical analyses and Sigmaplot was used to construct graphs. P values of less than 0.05 were considered significant.

## Supplementary information

Supplementary Figures.

Supplementary Information.
