# Supplementary material for: Age, Sex and Overall Health, Measured As Frailty, Modify Myofilament Proteins in Hearts From Naturally Aging Mice
Source: Sci Rep. 2020 Jun 22;10:10052. doi: 10.1038/s41598-020-66903-z (PMC7308399; doi:10.1038/s41598-020-66903-z)

SUPPLEMENTARY FILES

AGE, SEX AND OVERALL HEALTH, MEASURED AS FRAILTY, MODIFY MYOFILAMENT PROTEINS IN HEARTS FROM NATURALLY AGING MICE

Alice E Kane[1,2], Elise S Bisset[2], Kaitlyn M Keller[2], Anjali Ghimire[2],

W Glen Pyle[3,4] and Susan E Howlett[2,5]

Affiliations

[1]Blavatnik Institute, Department of Genetics, Paul F. Glenn Center for Biology of Aging Research at Harvard Medical School, Boston, MA

[2]Department of Pharmacology, Dalhousie University, Halifax, NS, Canada

[3]Department of Biomedical Sciences, University of Guelph, Guelph, Ontario, Canada

[4]IMPART team Canada Investigator Network

[5]Department of Medicine (Geriatric Medicine), Dalhousie University, Halifax, NS, Canada

Corresponding author

Susan Howlett, PhD

Department of Pharmacology

Sir Charles Tupper Medical Building

PO Box 15000

Halifax, Nova Scotia

Canada B3H 4R2

Phone: +1 902 494 3552

Email: susan.howlett@dal.ca

Running title: Sex and frailty modify myofilament proteins in aging

Supplementary Figure 1

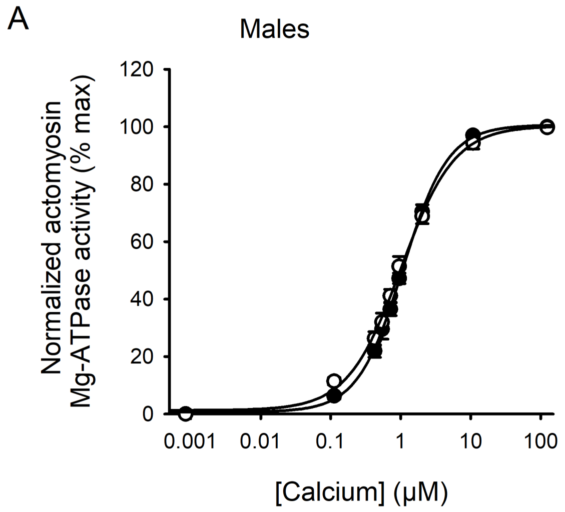

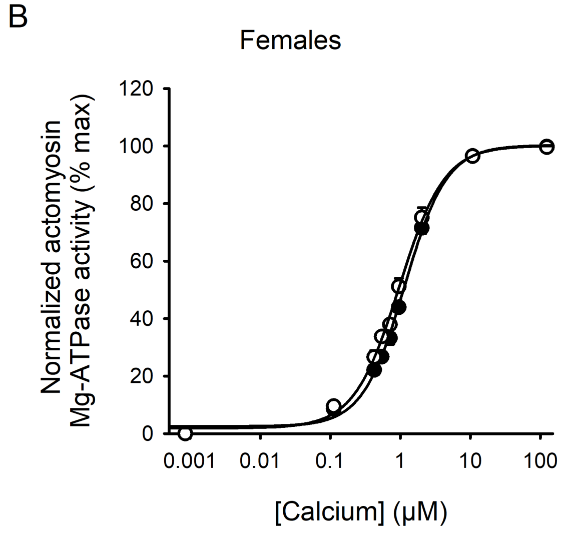

Supplementary Figure 2

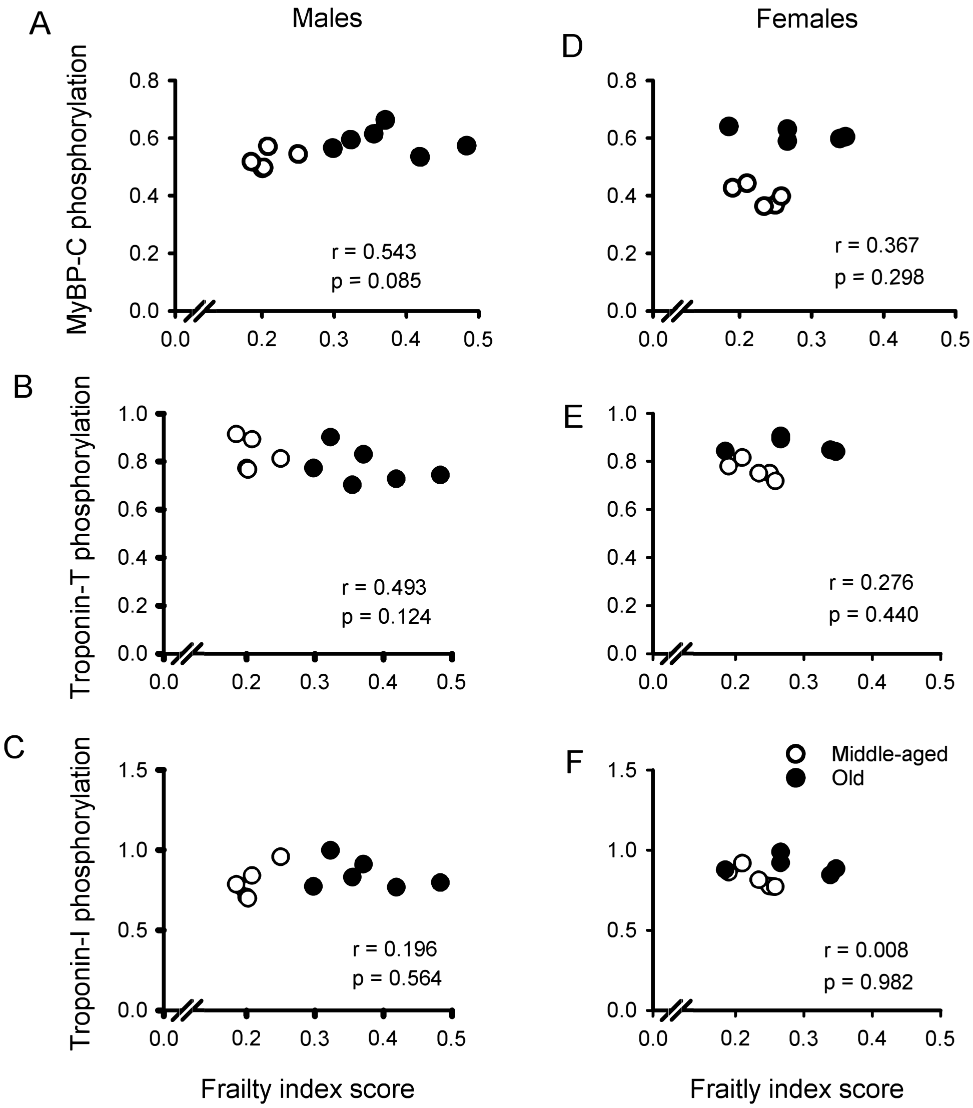

Supplement: Supplementary file 1 — Supplementary Figures. [file 41598_2020_66903_MOESM1_ESM.pdf]
