# Supplementary material for: Age, Sex and Overall Health, Measured As Frailty, Modify Myofilament Proteins in Hearts From Naturally Aging Mice
Source: Sci Rep. 2020 Jun 22;10:10052. doi: 10.1038/s41598-020-66903-z (PMC7308399; doi:10.1038/s41598-020-66903-z)
Supplement: Supplementary file 2 — Supplementary Information. [file 41598_2020_66903_MOESM2_ESM.pptx]

## Slide 1
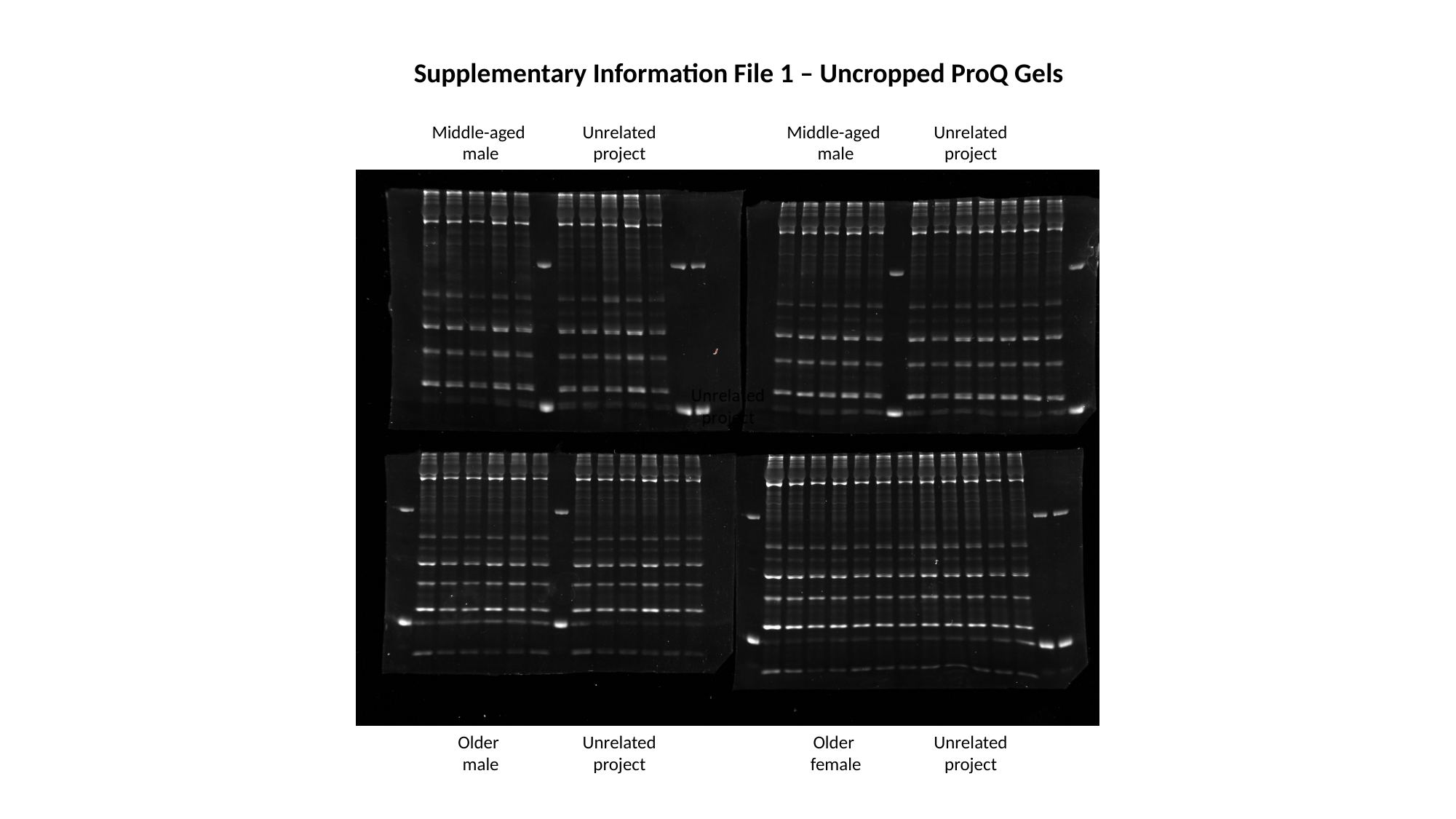

Supplementary Information File 1 – Uncropped ProQ Gels
Middle-aged
 male
Unrelated project
Middle-aged
 male
Unrelated project
Older
 male
Unrelated project
Older
 female
Unrelated project

## Slide 2
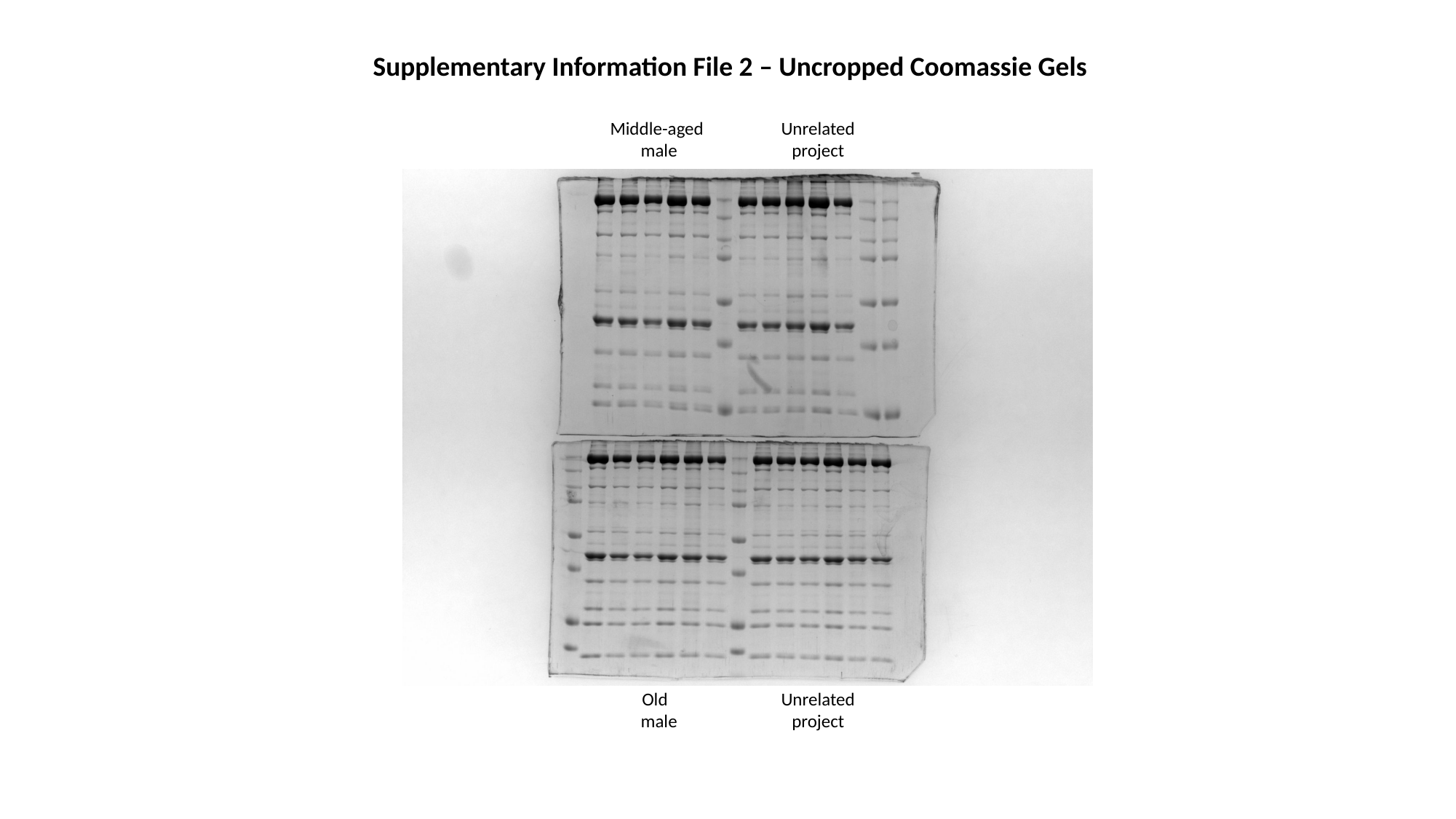

Supplementary Information File 2 – Uncropped Coomassie Gels
Middle-aged
 male
Unrelated project
Old
 male
Unrelated project

## Slide 3
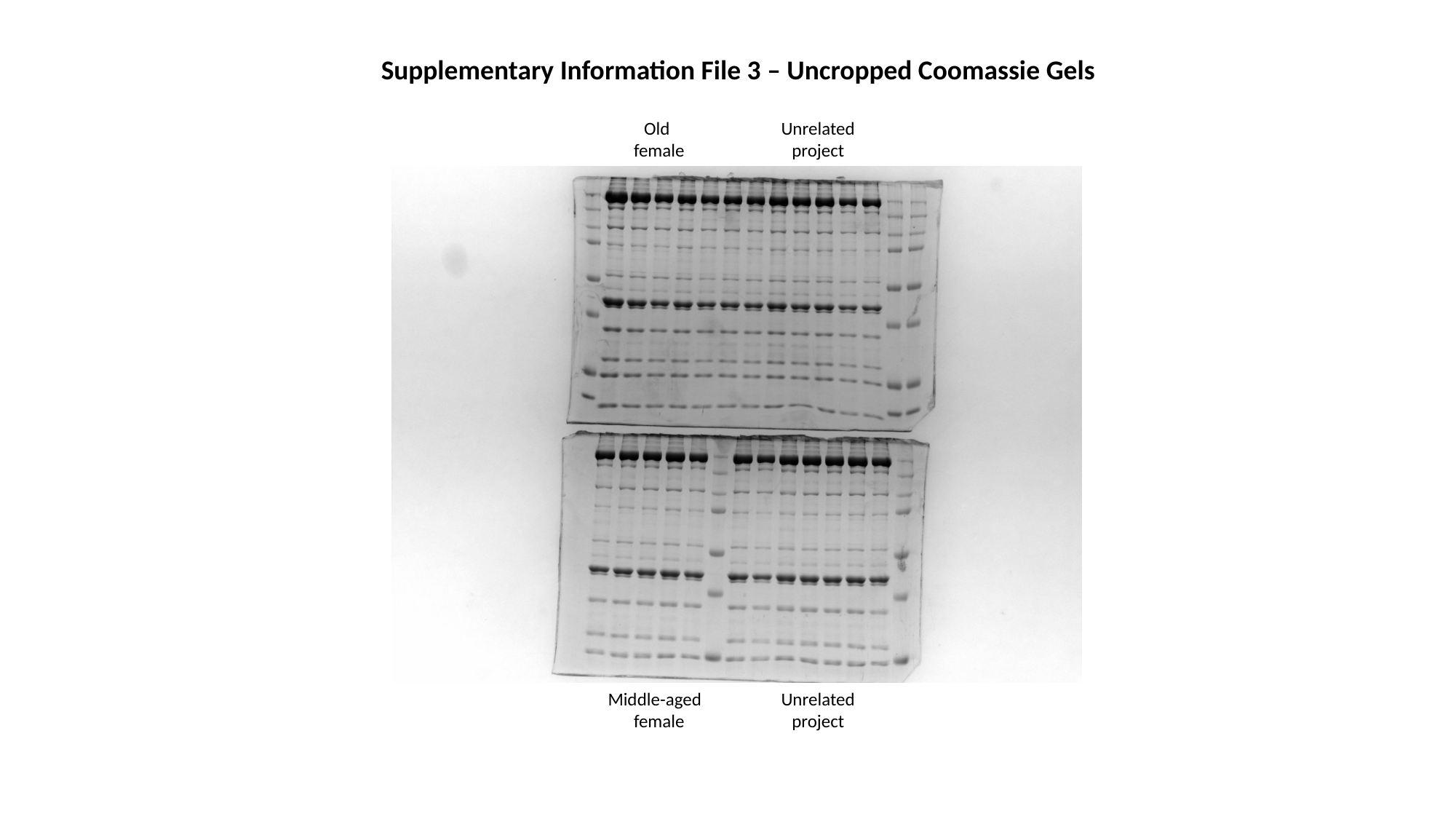

Supplementary Information File 3 – Uncropped Coomassie Gels
Old
 female
Unrelated project
Middle-aged
 female
Unrelated project
